# Exploring current evidence on bispecific CAR-T cell therapy for acute leukemias: a systematic review

**DOI:** 10.3389/fonc.2026.1720483

**Published:** 2026-04-23

**Authors:** Gabriela Valencia Putri Husodho, Anatalya Diah Ayu Kumalasari, Agyta Hanifa Faiza, Edward Kurnia Setiawan Limijadi, Meita Hendrianingtyas

**Affiliations:** 1 Faculty of Medicine, Universitas Diponegoro, Semarang, Indonesia; 2 Department of Clinical Pathology, Faculty of Medicine, Universitas Diponegoro, Semarang, Indonesia

**Keywords:** bispecific CAR-T cell, chimeric antigen receptor, dual-targeting, immunotherapy, leukemia

## Abstract

**Introduction:**

Chimeric antigen receptor T cell (CAR-T cell) therapy is an immunotherapy for acute leukemias utilizing recombinant receptors specific to antigens involved in T-cell function to direct tumor cell recognition and elimination. However, major limitations include relapse due to antigen escape and tumor heterogeneity, as well as on-target off-tumor effects. Bispecific CAR-T cells are developed to overcome these issues.

**Method:**

A literature search was conducted to identify relevant articles using three databases (PubMed, Scopus, ProQuest) between 2016 and 2025. Studies written in English and *in vivo*, *in vitro*, and clinical trial research designs were included. Articles without complete data and case reports were excluded. The systematic review followed PRISMA guidelines.

**Results:**

Nine studies were included in the final synthesis. Bispecific CAR-T cell therapy was superior in tumor eradication and limiting adverse effects in both AML and ALL. CARs used in AML often targeted CD123 and CD33; development of more diverse targets, including cell signaling of tumor growth (FLT3, NKG2D, TIM3, FRβ), addressed heterogeneity. CD19 was the most common CAR in ALL, additionally targeting CD22, CD20, and BAFF-R; addressing CD19-negative relapse was quite common. Phase I clinical trials were done for CD19/CD22 bispecific CAR-T cells, proving effective with low incidence of cytokine release syndrome, neurotoxicity, or other adverse effects. Preclinical trials showed that bispecific CAR-T cells had longer *in vivo* persistence.

**Conclusion:**

Bispecific CAR-T cell therapy is more effective in managing acute leukemias than conventional CAR-T cells. Future research should focus on developing diverse targets and advancing into clinical trials.

**Systematic Review Registration:**

Open Science Framework (OSF) Registries https://osf.io/, registration DOI: https://doi.org/10.17605.

## 1 Introduction

Acute leukemia is defined as the abnormal production of leukocytes from the bone marrow and can be classified based on its cell origin into acute myeloid leukemia (AML) and acute lymphoblastic leukemia (ALL) (1). AML causes rapid uncontrolled proliferation of myeloid progenitor cells, while ALL affects B or T lymphocytes along with lymphoid progenitor cells (2). ALL makes up around 25% of pediatric cancers, while AML is more often found in adults (3). However, the prevalence of ALL still reaches 15-30% in adults and increases with age due to the occurrence of relapse (2). The current management generally used for both leukemias is the use of chemotherapy, such as the ‘7 + 3 regimen’ for AML, but this itself has limitations in which side effects, drug resistance, and relapses are at high risk to occur (1, 4). The prevalence of leukemia relapse in adults further complicates the side effects of standard chemotherapy, as older patients tend to present with comorbidities (2). Recently, targeted immunotherapy is being developed to offer a more personalized approach to the patients’ disease, aiming to increase efficacy and reduce side effects, one of which includes chimeric antigen receptor (CAR)-based therapies (4).

Chimeric antigen receptors (CARs) themselves are recombinant receptors specific to antigens involved in the function of immune cells, most often T lymphocytes; these CARs can be used to target tumor-associated antigens, hence directing T cells to kill tumor cells, as leukemic cells tend to express receptors that respond to T cells (such as TCR) (5, 6). Blood is collected from cancer patients, then its T cells are extracted and modified to express CARs, developing CAR-T cells, which are then infused back into the patient (7). Patients undergo chemotherapy that aims to deplete existing lymphocytes, also known as the lymphodepletion stage, before the infusion of CAR-T cells to increase their effectiveness (8). The most common target for CAR-T cells is CD19 antigens found in B-ALL as well as other blood cancers, including B-NHL; this has been proven effective and has also been FDA-approved (9). However, a limitation to this therapy is the incidence of antigen loss or escape, resulting in CD19-negative relapses in around 20% of B-ALL as well as B-NHL patients, hence becoming treatment resistant (10, 11). Even though 70-94% of patients achieve complete remission (CR), relapse is still commonly observed due to failure of CAR-T cell expansion or limited persistence (12). The heterogeneity of AML also contributes to low response rates towards CAR-T cell therapy (13). Therefore, more various targets for CARs need to be developed to increase efficacy and address AML and ALL relapse after therapy.

**Figure 1:**
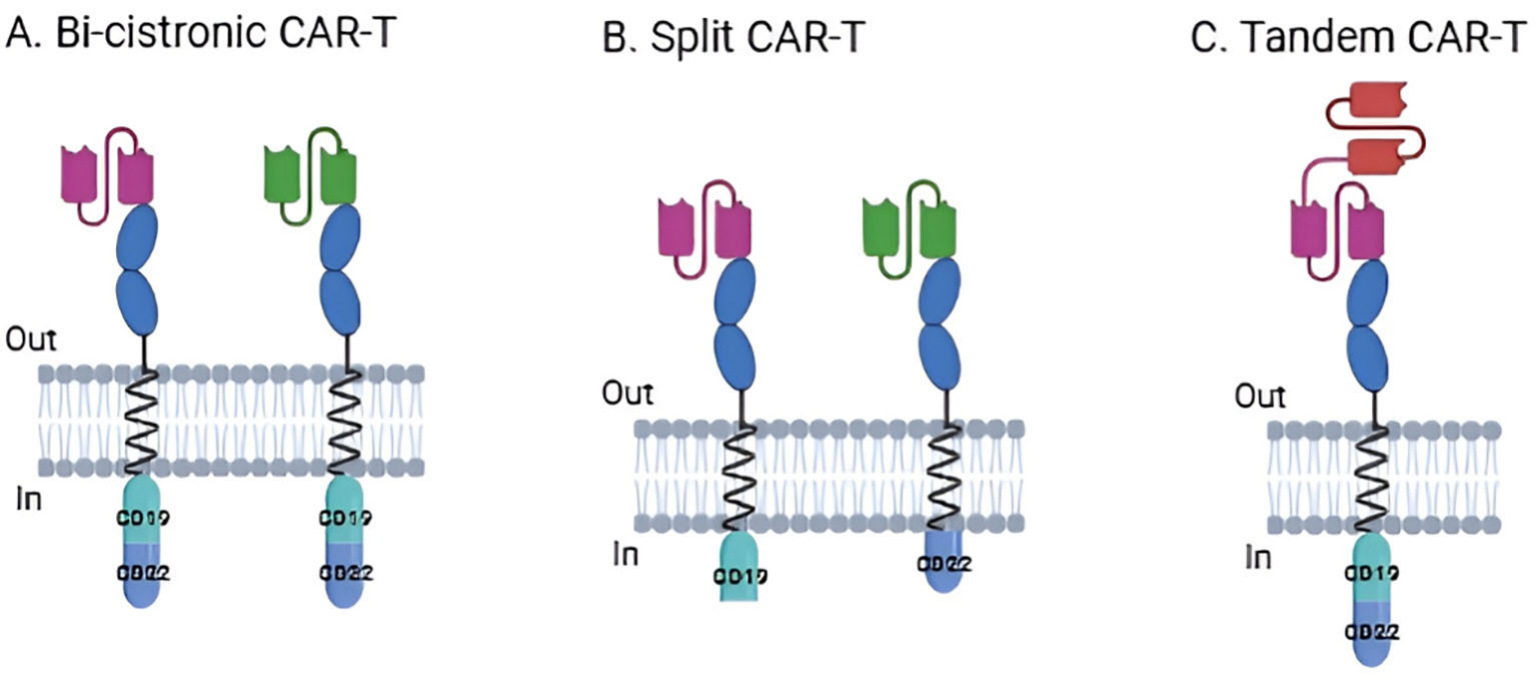
Bispecific CAR types based on engineered construct. **(A)** Bi-cistronic CAR-T. **(B)** Split CAR-T. **(C)** Tandem CAR-T.

Recently, bispecific or dual-targeting CAR-T cells are being developed, hence increasing efficacy and overcoming evasion mechanisms (14). These T cells are engineered to express more than one CAR, enabling simultaneous recognition of two tumor-associated antigens. CAR-T cells that have more than one target can be classified based on their engineering (**Figure 1**), into bicistronic (which have two CARs that target antigens independently), tandem (a CAR with two targets fused into one construct), and split CARs (which target two co-stimulatory domains independently) (15, 16). This concept of dual targeting has been applied to other immunotherapies before, such as with T cell engagers and bispecific antibodies, but bispecific CAR-T cell therapy differs in that both CARs target, not only engage, two distinct antigen targets (17). Solid tumors are most often the target of this therapy due to their heterogeneity, but acute leukemia can also benefit from this, as antigen escape is a common cause of relapse (17, 18). This systematic review intends to discuss advancements regarding bispecific CAR-T cell therapy, from *in vitro* and *in vivo* studies of developing novel target combinations to clinical trials of approved therapies, as a next step in the era of personalized medicine.

## 2 Methods

### 2.1 Study design

This study was a systematic review conducted to synthesize existing evidence on the efficacy of CAR-T cell-based therapy in the treatment of acute leukemias. This design was chosen as it allows for comprehensive identification, analysis, and evaluation of relevant studies, providing a robust foundation for understanding the topic. We conducted this systematic review from April 2025 to May 2025.

### 2.2 Research question

Our primary research question was formulated using the Population, Intervention, Comparison, and Outcome (PICO) framework (19). Population: ALL and AML patients, leukemic cell cultures or animal models with ALL or AML, Intervention: bispecific CAR-T cell, Comparison: control group (if applicable), Outcome: eradication of tumor cells, occurrence of adverse effects or toxicity. Our study was guided by the following research question: *how is the efficacy of bispecific CAR-T cell therapy in the treatment of acute lymphoblastic leukemia (ALL) and acute myeloid leukemia (AML)?*

### 2.3 Search strategy

A comprehensive search strategy was developed to capture a wide array of studies addressing this topic. The keyword string used for the literature search is as follows: “bispecific chimeric antigen receptor” OR “bispecific CAR-T cell therapy” OR “dual-targeting CAR” OR “tandem CAR,” “split CAR,”, “acute leukemia”, “ALL”, and “AML”. Boolean operators AND/OR were utilized to ensure precision and inclusivity. We searched three databases, including PubMed, Scopus, and ProQuest, to identify relevant literature. We employed the following inclusion and exclusion criteria in choosing the articles:

- Inclusion criteria.
  - Studies with *in vivo*, *in vitro*, and clinical trial research designs.
  - Articles published within the last 10 years, that is 2016-2025.
  - Articles written in English.

- Exclusion criteria.
  - Articles that do not have complete data or has not been published.
  - Articles in which the keywords do not match with the title and abstract.
  - Articles that cannot be fully accessed.

### 2.4 Screening of the article

All articles retrieved through database searches were imported into Rayyan.ai for consolidation and deduplication. Subsequently, independent reviewers among GVPH, ADAK, and AHF conducted a three-step screening process. 1) Title Screening: Titles were screened for relevance; 2) abstract screening: Abstracts were evaluated to confirm alignment with the inclusion criteria; 3) full-text screening: Eligible studies underwent thorough full-text analysis to finalize the selection. Discrepancies were resolved through discussion and consultation.

### 2.5 Data extraction

A standardized data extraction form agreed upon by the authors was used to systematically collect data from included studies. Data collected from the articles included the following: author, year of publication, target of CARs, methodology, leukemia type, research subject and sample, and result.

### 2.6 Quality appraisal of the included studies

**Figure 2:**
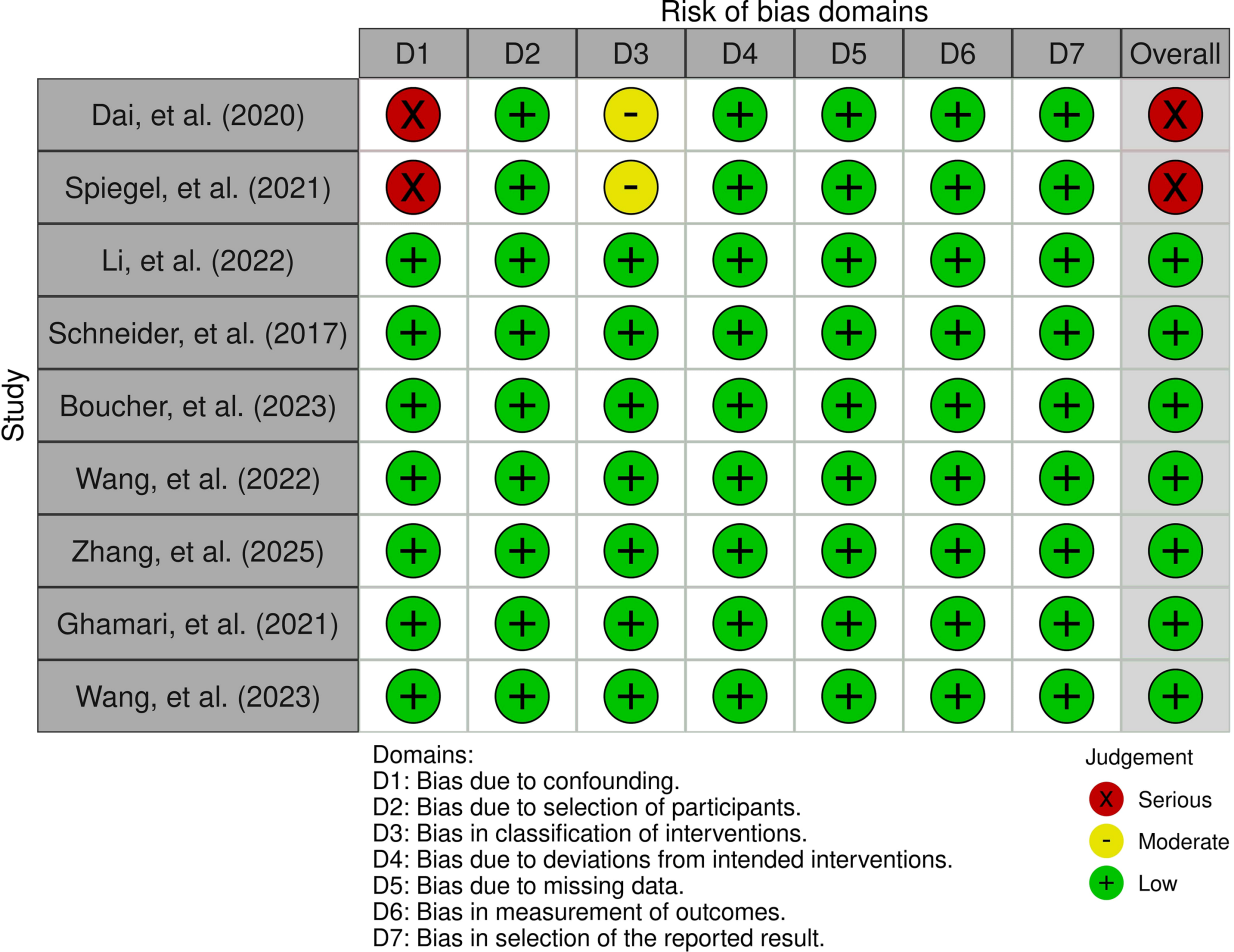
Traffic-light plot of ROBINS-I quality assessment.

**Figure 3:**
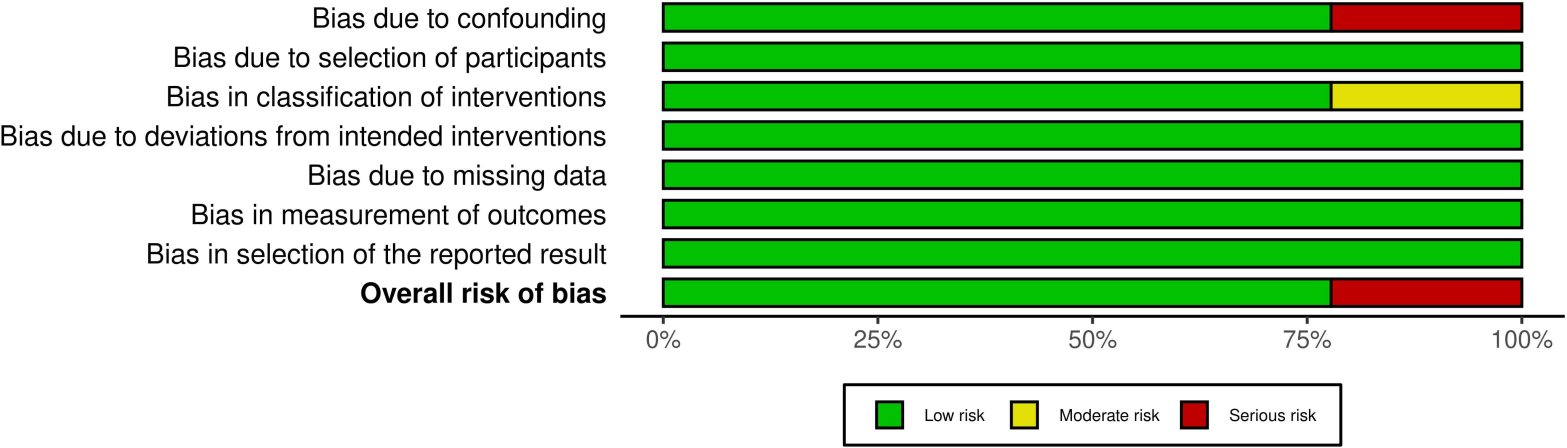
Summary plot of ROBINS-I quality assessment.

The risk of bias was assessed using the Risk of Bias in Non-randomized Studies of Interventions (ROBINS-I) tool. Two authors independently assessed the studies based on the tool criteria. Any discrepancies were resolved by discussion. In each study, bias was assessed in seven domains: confounding, classification of interventions, selection of participants, deviations from intended interventions, missing outcome data, outcome measurement, and selection of reported results. Bias in each domain was categorized as low, moderate, serious, or critical. Finally, the prior assessment results were used to determine the overall risk of bias in each study. The results of the quality appraisal are presented in **Figures 2**, **3**.

### 2.7 Data analysis

A conventional inductive content analysis approach was employed in the data analysis process, resulting in the decision to categorize results based on leukemia type (AML and AML). A descriptive approach was used to analyze the efficacy of bispecific CAR-T cells in the eradication of leukemia tumor cells as well as the occurrence of adverse effects.

### 2.8 Registration

The study was registered in Open Science Framework (OSF) Registries, with the approved registration DOI https://doi.org/10.17605/OSF.IO/BXJRQ.

## 3 Results

**Figure 4:**
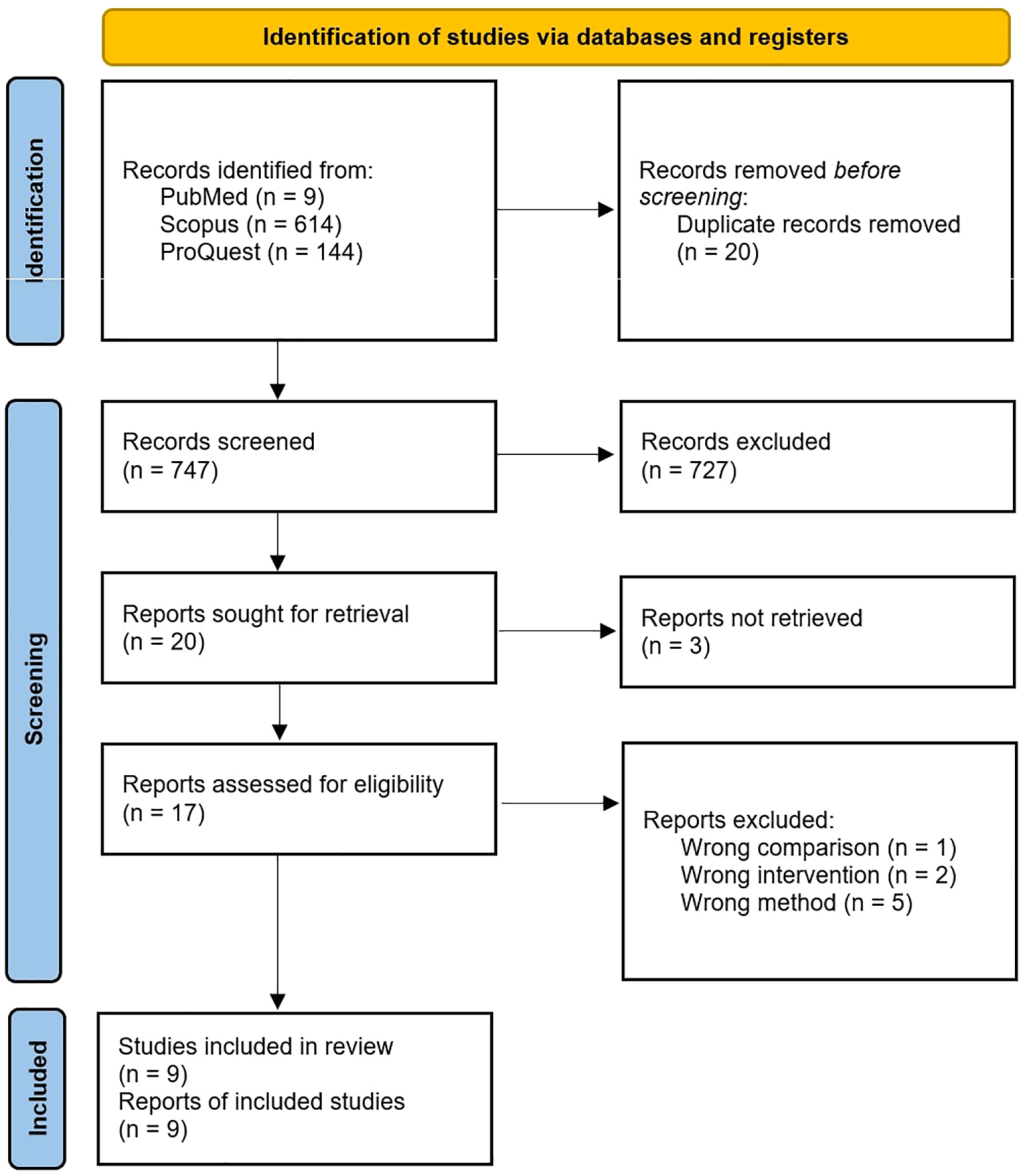
PRISMA of the search flow.

As per our study assessing the efficacy of bispecific CAR-T cell therapy for treatment of ALL and AML, a total of 767 articles were screened based on the inclusion and exclusion criteria. Out of the total reviewed articles, 9 articles were obtained as outlined in **Figure 4**. The data characteristics of included studies are described as in **Table 1**.

**Table 1:** Characteristics of included studies.

No	Author	Target	Method	Leukemia type	Sample
1	Dai, et al. (2020) (20).	CD19 and CD22	Phase I Clinical trial	B-ALL	Bone Marrow
2	Spiegel , et al. (2021) (21).	CD19 and CD22	Phase I Clinical trial	B-ALL	Bone Marrow
3	Li, et al. (2022) (22).	FLT3scFv/NKG2D	*In vitro* & *in vivo*	AML with FLT (FLTmut+) mutations	Bone marrow
4	Schneider, et al. (2017) (23).	CD19/CD20	*In vitro* and *In vivo*	ALL	*In vitro*: • NALM-6 (ALL cell line, CD19+CD22+, low CD20+) • REH (ALL cell line, CD19+CD20-CD22+) *In vivo*: Mouse-adapted Raji-luc cells
5	Boucher, et al. (2023) (24).	CD33/CD123	*In vivo*	AML	Xenograft AML mouse model (human AML-derived MV411 cells) and NSG mice engrafted with human CD34+ cells
6	Wang, et al. (2022) (25).	CD19/BAFF-R	*In vivo* and *in vitro*	ALL	*In vivo*: NSG mouse model of heterogenous leukemia (Nalm-6 CD19−/− and BAFF-R−/−)) *In vitro*: Primary ALL cells (NALM-6 cell lines)
7	Zhang, et al. (2025) (26).	CD13/TIM3	*In vivo* and *in vitro*	AML	*In vivo*: Xenograft AML mouse model, HIS mice injected CD34+ cells *In vitro*: Kasumi6 tumor cells
8	Ghamari, et al. (2021) (27).	FRβ and CD123	*In vitro*	B-AML	THP1 (12) and MV4–11 human AML cell lines
9	Wang, et al. (2023) (28).	CD123/CLL-1	*In vitro*	AML	THP-1 and K562 cell lines

### 3.1 Acute myeloid leukemia

Five preclinical studies were found that researched different bispecific CAR-T designs and demonstrated significant cytotoxic activities against AML both *in vitro* and *in vivo*, utilizing various antigen targets and engineering platforms (22, 24, 26–28). Most studies utilized CD34+ cells in *in vivo* studies, as research previously showed that these cell lines offer monitoring of treatment response in AML (29).

Li et al. (2022) investigated a dual-target approach combining bispecific FLT3scFv/NKG2D CAR-T cells with the FLT3 inhibitor gilteritinib in relapsed/refractory FLT3-mutated AML. *In vitro*, the bispecific CAR-T cells showed potent cytotoxicity against AML cells, with a notably higher efficacy towards FLT3mut+ cells. *In vivo*, monotherapy with CAR-T cells prolonged mouse survival, while gilteritinib alone extended survival. Remarkably, the combination therapy further improved survival to 35 days, alongside an increased distribution of CAR-T cells in the bone marrow, supporting a synergistic anti-leukemic effect (22).

Boucher et al. (2023) explored bispecific CD33/CD123 CAR-T cells using an “AND gate” split signaling design to target dual-antigen AML blasts. Utilizing a xenograft mouse model with human MV411 cells and NSG mice engrafted with CD34+ cells, they demonstrated that the bispecific CAR-T cells effectively controlled AML progression. Importantly, these CAR-T cells exhibited minimal off-tumor toxicity, showing low cytotoxicity toward healthy CD34+ hematopoietic progenitors, addressing one of the major limitations observed in monospecific CD33- or CD123-targeted therapies (24).

Similarly, Zhang et al. (2025) developed bispecific CAR-T cells targeting CD13 and TIM-3. The novel split-signaling architecture combined anti-CD13 and anti-TIM3 nanobodies linked to distinct intracellular signaling modules. *In vitro*, the nbiCARTs demonstrated strong cytotoxic effects against Kasumi6 tumor cells expressing both CD13 and TIM-3. In xenograft models with humanized immune systems (HIS mice), the nbiCARTs eradicated AML tumors without significant damage to bone marrow-derived colony-forming progenitors or human CD34+ HSCs. These findings highlighted the potential of dual-antigen targeting in reducing relapse risk while preserving normal hematopoiesis (30).

To address tumor antigen loss and heterogeneity, Ghamari et al. (2021) developed a bispecific tandem CAR-T construct targeting CD123 and folate receptor beta (FRβ) for B-AML. Using THP1 and MV4–11 AML cell lines *in vitro*, their TanCAR-T cells achieved superior tumor lysis compared to monospecific CARs beyond 72 hours of culture. The TanCAR-T cells induced significantly higher secretion of IFN-γ and IL-2, reflecting enhanced T cell activation and persistence, thus offering a robust strategy against antigen escape (27).

Finally, Wang et al. (2023) constructed tandem bispecific CAR-T cells targeting CD123 and CLL-1. *In vitro* experiments using THP-1 and K562 cell lines revealed that tandem CAR-T cells retained high cytotoxicity against single-antigen-expressing targets but exhibited significantly superior killing against dual-antigen tumor cells compared to single-target CAR-T cells. Additionally, tandem CAR-T cells released higher levels of proinflammatory cytokines, emphasizing their enhanced immune activation profile and potential to combat the diverse antigenic landscape characteristic of refractory AML (28).

### 3.2 Acute lymphoblastic leukemia

Four studies regarding bispecific CAR-T cell therapy for ALL were found; two were preclinical studies, and two were clinical trials (20, 21, 23, 25).

Wang, et al. in 2022 had developed a dual-targeting CAR-T cell approach to address antigen escape in ALL treatment, utilizing the loop configuration (CD19-BAFF-R(l)), which demonstrated superior efficacy. These CAR-T cells exhibited antigen-specific cytokine release, degranulation, and cytotoxicity against CD19−/− and BAFF-R−/− variant human ALL cells *in vitro*. In immunodeficient NSG mouse models with mixed antigen-negative variants, a single dose of CD19-BAFF-R dual CAR-T cells was able to completely eradicate both variants. These also showed prolonged *in vivo* persistence compared to monospecific CD19-CAR-T cells. These findings demonstrate that simultaneous targeting of CD19 and BAFF-R can overcome antigen escape, potentially improving remission durability in ALL treatments. The research supports clinical translation of this dual-targeting approach for treating heterogeneous B-cell malignancies (25).

Research conducted by Schneider, et al. in 2017 focused on developing tandem CAR-T cells targeting CD19 and CD20 antigens to combat tumor antigen escape mechanisms in leukemia. The tandem CAR construct exhibited superior performance, with enhanced cytolytic activity against leukemia cells, intermediate cytokine production (IFNγ, TNFα, IL-2, and GM-CSF), and a remarkable ability to maintain immune pressure on target cells. *In vitro* and *in vivo* experiments revealed the tandem CARs’ ability to eliminate leukemia cells, effectively controlling tumor growth without significant toxicity. The research highlighted the potential of tandem CARs to mitigate antigen escape mechanisms by targeting multiple tumor antigens simultaneously (23).

Only CD19/CD22 targeting bispecific CAR-T cells have been used in human subjects. Two phase I clinical trials have been done for CD19/CD22 CAR-T cell therapy, one of which was reported by Dai, et al. (2020). Six adult patients who received autologous CD19/CD22 CAR-T cells at doses ranging from 1.7×10^6 to 3×10^6 CAR-T cells per kilogram of body weight following lymphodepleting chemotherapy. The results showed that bispecific CD19/CD22 CAR-T cells triggered robust cytolytic activity against target cells, with all 6 patients achieving minimal residual disease-negative complete remission (MRD-negative CR). The CAR-T cells proliferated *in vivo* and were detected in blood, bone marrow, and cerebrospinal fluid. No neurotoxicity occurred in any patients, though all experienced cytokine release syndrome (CRS) (grades 1–2 were observed, and grade ≥3 was not reported). Three patients relapsed at 3-, 5-, and 10-months post-treatment, with one patient showing antigen escape through CD19 loss and diminished CD22 expression and the other two patients having antigen-positive relapse associated with loss of CAR-T cell persistence. Three patients maintained ongoing remission, with two patients in continuous remission for more than 8 months at the time of reporting (20).

In 2021, Spiegel, et al. conducted a phase I clinical trial that investigated a bispecific CAR targeting both CD19 and CD22 in adult patients with relapsed/refractory B-ALL and large B-cell lymphoma (LBCL). The study enrolled 39 patients (17 with B-ALL, 22 with LBCL), with 38 receiving the CAR T-cell infusion after conditioning chemotherapy. The researchers demonstrated successful manufacturing, with 97% of products meeting protocol-specified dose requirements and no dose-limiting toxicities during dose escalation. CRS occurred in 76% of patients (with mostly grade 1-2, and only 1 patient had grade ≥3 CRS), and neurological toxicity occurred in 37%. In B-ALL patients, the response rate was 100%, with 88% (15 out of 17 patients) achieving minimal residual disease-negative complete remission (CR). Durable MRD-negative status was observed in most responders, indicating deep molecular remission. For LBCL patients, the response rate was 62% with 29% CR. Importantly, relapses were frequently associated with CD19-/lo disease (50% in B-ALL and 29% in LBCL) but were not associated with CD22-/lo disease (21).

## 4 Discussion

### 4.1 Treatment efficacy

The main limitation with conventional monospecific CAR-T cell therapy is due to antigen expression heterogeneity in AML, leading to antigen escape and on-target off-tumor toxicities (13, 31). In accordance with that, the targets being developed for AML are quite varied. CD33 is expressed in 85–90% of adult and pediatric AML, making it a suitable target, and has one article that utilized this target to make CD33/CD123 CAR-T cells (24, 32). CD123 was also used as a target in three studies (24, 27, 28). The heterogeneity of AML opens up opportunities for a wider range of targets other than CDs, including FLT3, NKG2D, TIM3, and FRβ, that were studied in three studies (22, 27, 30). Instead of only targeting tumor surface antigens, these CAR-T cells also target receptors involved in the expansion of AML tumor cells, overcoming the disease through another perspective. Collectively, the six studies regarding AML bispecific CAR-T cell therapy demonstrate that bispecific CAR-T cells, regardless of their CAR construct, markedly improve the specificity and potency of CAR-T therapies against AML. The integration of novel engineering approaches has successfully minimized off-tumor toxicity while maintaining strong anti-leukemic activity, paving the way for clinical trials aimed at validating their safety and efficacy in human patients.

All studies regarding bispecific CAR-T cells developed for ALL found in this review targeted CD19 as one of their antigens, as CD19 is found in essentially most lymphoblastic leukemias (33). However, CD19 also tends to undergo antigen escape in relapse, resulting in CD19-negative relapses in ALL patients; therefore, the development of bispecific CAR-T cells is beneficial in this context (10). CD19/CD22 bispecific CAR-T cells have been approved to undergo phase I clinical trials by Dai, et al. and Speigel et al. in 2020 and 2021 respectively (20, 21). One limitation sometimes found in CAR-T cell therapy is the occurrence of CRS which is the consequence of rapid immune activation and can be life-threatening (34). In the clinical trials of CD19/CD22 bispecific CAR-T cells, the majority of patients did not experience CRS or only experienced grade 1–2 CRS if they did; the occurrence of grade 3 CRS was uncommon and was easily manageable with drugs (20, 21, 35). The same was seen with neurotoxicity as a side effect of the therapy, with no neurotoxicity found in the trial done by Dai, et al. (2020) and only a small percentage of patients developing neurotoxicity that was reversible in the trial done by Spiegel, et al. (2021) (20, 21). CAR-T cell targets being developed in preclinical studies include CD20 and BAFF-R, with CD19/CD20 CAR-T cells seen having higher efficacy and lower toxicity compared to single-targeting CARs, and CD19/BAFF-R dual-targeted CAR T cells having increased *in vivo* persistence (23, 25). Overall, these four studies concluded that the advancements of bispecific CAR-T cell therapy for ALL have come close to being approved in clinical settings, as well as resulting in lower toxic effects and having higher *in vivo* persistence compared to conventional monospecific CAR-T cells.

### 4.2 Safety aspects

Bispecific CAR-T cell therapies have emerged as a promising approach to treating hematologic malignancies such as acute myeloid leukemia (AML) and acute lymphoblastic leukemia (ALL) by simultaneously targeting two antigens, which reduces antigen escape and improves efficacy. However, despite their potential, these therapies raise safety concerns primarily related to immune-related toxicities, including CRS and neurotoxicity.

One of the key safety advancements in bispecific CAR-T therapy is the reduction of off-tumor toxicity, a significant challenge in traditional monospecific CAR-T therapies. For example, bispecific CAR-T cells targeting CD33/CD123 in AML have shown selective cytotoxicity, effectively targeting leukemic cells while sparing normal hematopoietic stem and progenitor cells (HSPCs), reducing the risk of myelotoxicity. This split-signaling design in these cells requires both antigens to be present for full T-cell activation, minimizing off-target effects (24). Similarly, bispecific CD13/TIM-3 CAR-T cells have demonstrated strong anti-leukemic activity in preclinical models without harming normal bone marrow progenitors. The use of dual-targeting CAR-T cells offers an important strategy to improve selectivity and reduce collateral damage to healthy tissue (26). Moreover, combining bispecific CAR-T cells with small molecules such as gilteritinib in FLT3-mutated AML has shown synergistic effects, enhancing T-cell infiltration and survival without evidence of increased toxicity in reported models (22).

Despite these innovations, CRS remains a major concern in CAR-T therapies. CRS is characterized by fever, hypotension, and elevated cytokines such as IL-6 and IL-10, and it is commonly observed in clinical trials targeting CD19/CD22 in relapsed/refractory ALL. While most CRS cases are low-grade (1–2), severe cases can occur but are usually manageable with supportive care or tocilizumab administration (36). In AML, the risk of severe CRS varies depending on the CAR construct; for example, bispecific CD123/CLL-1 CAR-T cells exhibit potent cytokine secretion and proliferation, raising concerns about CRS, but these risks can be mitigated with anti-IL-6 agents (37).

Neurotoxicity, or immune effector cell-associated neurotoxicity syndrome (ICANS), is another potential adverse event. While relatively infrequent, neurotoxicity can occur, and cases typically resolve with corticosteroid treatment (38). The safety profile of bispecific CAR-T therapies also depends on antigen expression patterns. CD123, while highly expressed on AML blasts, is also present on normal hematopoietic progenitors and endothelial cells, which can lead to off-tumor toxicity. Using split-signaling CAR-T cells targeting CD33/CD123 has shown reduced toxicity to healthy CD34+ hematopoietic stem cells compared to monospecific CAR-T cells (39, 40).

### 4.3 Limitations and recommendations for future studies

Among the 10 studies reviewed, 8 employed a preclinical trial design, and 2 were phase I clinical trials. There is still limited research on the applications of bispecific CAR-T cell therapy for leukemia in human subjects; thus, furthering the phases of clinical trials is recommended for future studies. The clinical trials also did not use control groups, impacting the risk of bias of those studies. The only bispecific CAR-T cells that have been used so far in humans are the CD19/CD22 T cells; further development of more diverse targets is recommended for future studies to overcome adverse effects of bispecific CAR-T cells that have not been approved for clinical trials, as well as to increase their effectiveness in both initial usage and overcoming or preventing relapse.

## 5 Conclusion

Bispecific CAR-T cell therapy is superior in terms of acute leukemia (both ALL and AML) treatment, as they can overcome antigen loss and heterogeneity and have fewer on-target off-tumor effects than conventional single-targeting CAR-T cells. Current evidence reveals only limited bispecific CAR-T cells have entered clinical trials; thus, future research should focus on developing more diverse targets to increase efficacy and decrease adverse effects of this therapy so that it can be used in clinical settings. Other therapy aspects, such as manufacturing complexity and high cost, should also be acknowledged to increase accessibility.

## Data Availability

The original contributions presented in the study are included in the article/**Supplementary Material**. Further inquiries can be directed to the corresponding author.
