# Supplementary material for: Exploring current evidence on bispecific CAR-T cell therapy for acute leukemias: a systematic review
Source: Front Oncol. 2026 Apr 23;16:1720483. doi: 10.3389/fonc.2026.1720483 (PMC13149160; doi:10.3389/fonc.2026.1720483)
Supplement: Supplementary file 1 [file Table1.docx]

Supplementary Material

##

Table 1. Summary of articles and results

| **No.** | **Author** | **Target** | **Method** | **Leukemia Type** | **Sample** | **Result** |
| --- | --- | --- | --- | --- | --- | --- |
| 1 | Dai, et al. (2020).^24^ | CD19 and CD22 | Clinical trial | B-ALL | Peripheral Blood, Bone Marrow Aspirate, Cerebrospinal Fluid | - All 6 adult patients with relapsed/refractory B-ALL achieved complete remission (CR) with MRD-negativity following bispecific CD19/CD22 CAR-T infusion. - Only Grade 1–2 Cytokine release syndrome (CRS) observed. - No neurotoxicity was observed - Dual CD19/CD22-targeted CAR-T therapy is safe, effective, and potentially reduces the risk of antigen-negative relapse in adult R/R B-ALL. |
| 2 | Spiegel , et al. (2021).^25^ | CD19 and CD22 | Clinical trial | B-ALL | Peripheral Blood, Bone Marrow Aspirate, *In vitro* stimulation assay | - All patients with relapsed/refractory B-ALL achieved a clinical response after infusion with CD19/CD22 bispecific CAR-T cells. - 88% patients achieved CR with minimal residual disease (MRD) negativity. - Durable MRD-negative status was observed in most responders, indicating deep molecular remission. - Median overall survival (OS): 11.8 months - Median progression-free survival (PFS): 5.8 months - Relapse occurred in 10 patients, with:   - 5 showing CD19− or CD19-low leukemia at the time of relapse   - CD22 expression preserved in all relapsed cases - Cytokine release syndrome (CRS) occurred in 76% of patients, mostly Grade 1–2. - Neurotoxicity was observed in some patients, but was generally manageable and fully reversible. |
| 3 | Li, et al. (2022).^18^ | FLT3scFv/NKG2D | *In vitro* & *in vivo* | AML with FLT (FLTmut+) mutations | Bone marrow | *In vitro* :   - CAR-T FLT3scFv/NKG2D showed significant cytotoxicity towards AML cells - CAR-T cells were effective towards both FLT3mut+ and FLTmut- cells with higher efficacy towards FLT3mut+ cells - Increased secretion of IL-2 and IFN-γ which showed T cell activation   *In vivo* :   - CAR-T monotherapy prolonged survival compared to control group (24 vs 15 days) - Gilteritinib monotherapy improved survival rates (19 vs 15 days) - Combination therapy was significantly more effective (35 vs 24 days) - Increased CAR-T distribution in bone marrow in combination therapy group |
| 4 | Schneider, et al. (2017).^26^ | CD19/CD20 | *In vitro* and *In vivo* | ALL | *In vitro*:   - NALM-6 (ALL cell line, CD19+CD22+, low CD20+) - REH (ALL cell line, CD19+CD20-CD22+)   *In vivo*:  Mouse-adapted Raji-luc cells | - CAR Expression: Both tandem CAR constructs were successfully expressed on T cell surface (61-93%) and capable of binding target antigens - Cytolytic Activity: Tandem CARs demonstrated cytolytic activity against leukemia cells expressing CD19 and/or CD20, with CAR2019 showing better activity than CAR1920 - Cytokine Production: Tandem CARs produced IFNγ, TNFα, IL-2, and GM-CSF at intermediate levels between single CD19 CAR and single CD20 CAR - *In vivo* Activity: Tandem CARs effectively eliminated leukemia cells in mouse models, equivalent to single CD20 CAR and superior to single CD19 CAR - Antigen Escape: In co-culture with CAR-T, Raji cells rapidly downregulated CD19 expression, while CD20 expression remained relatively stable - High Tumor Burden Model: Tandem CAR 2019 showed efficacy without toxicity, while combination of CD19+CD20 CARs showed high toxicity |
| 5 | Boucher, et al. (2023).^20^ | CD33/CD123 | *In vivo* | AML | Xenograft AML mouse model (human AML-derived MV411 cells) and NSG mice engrafted with human CD34+ cells | CD33/CD123 bispecific CAR T cells were able to control AML in a xenograft AML mouse model similar to monospecific CD33 and CD123 CAR T cells while showing no on-target off-tumor effects (lower cytotoxicity towards CD34+ cells). |
| 6 | Wang. et al. (2022).^27^ | CD19/BAFF-R | *In vivo* and *in vitro* | ALL | *In vivo*: NSG mouse model of heterogenous leukemia (Nalm-6 CD19−/− and BAFF-R−/−))  *In vitro*: Primary ALL cells (NALM-6 cell lines) | *In vivo*:   - Superior antitumor activity of CD19-BAFF-R(l) dual CAR T cells over monospecific CD19 and BAFF-R CAR T cells, which led to significantly improved mouse survival. - Post CAR-T cell therapy, there was significant reduction in CD10+ leukemic cells in mice treated with dual versus monospecific CD19CAR T cells   *In vitro*:   - CAR T cells had significantly superior cytotoxicity against NALM-6 cell lines compared to mock T cells |
| 7 | Zhang, et al. (2025).^28^ | CD13/TIM3 | *In vivo* and *in vitro* | AML | *In vivo*: Xenograft AML mouse model, HIS mice injected CD34+ cells  *In vitro*: Kasumi6 tumor cells | - nbiCARTs showed strong antitumor activity to CD13/TIM3 positive leukemic cells *in vitro* and in preclinical models. - nbiCARTs had little toxicity to human bone marrow-derived colony forming progenitors ex vivo and the human HSCs in mice with a humanized immune system. |
| 8 | Ghamari, et al. (2021).^22^ | FRβ and CD123 | *In vitro* | B-AML | THP1 (12) and MV4-11 human AML cell lines | - At first, all CAR-T cell products (monospecific and TanCAR-T) significantly lysed the tumor cell lines. At and beyond 72 hours, bispecific TanCAR-T cells were significantly better able to cytolyze tumor cell lines compared with the other cells - TanCAR-T cell significantly induced IFNγ and IL-2 production more than single CAR-T cells, |
| 9 | Wang, et al. (2023).^23^ | CD123/CLL-1 | *In vitro* | AML | THP-1 and K562 cell lines | - Killing efficiency of tandem CAR-T cells in the case of single antigen expression is comparable to that of single target CAR-T cells.   Dual target CAR-T cells significantly surpass single target CAR-T cells in dual target tumor cells,   - CD123/CLL-1 CAR-T cells in tandem targeted and killed CD123- and CLL-1-positive leukemia cell lines and released a large number of cytokines. |
